# Rescue of Amyloid-Beta-Induced Inhibition of Nicotinic Acetylcholine Receptors by a Peptide Homologous to the Nicotine Binding Domain of the Alpha 7 Subtype

**DOI:** 10.1371/journal.pone.0067194

**Published:** 2013-07-22

**Authors:** Arthur A. Nery, Margaret H. Magdesian, Cleber A. Trujillo, Luciana B. Sathler, Maria A. Juliano, Luiz Juliano, Henning Ulrich, Sergio T. Ferreira

**Affiliations:** 1 Department of Biochemistry, Chemistry Institute, São Paulo University, São Paulo, SP, Brazil; 2 Institute of Medical Biochemistry, Federal University of Rio de Janeiro, Rio de Janeiro, RJ, Brazil; 3 Department of Biophysics, Federal University of São Paulo, São Paulo, SP, Brazil; University G. D'Annunzio, Italy

## Abstract

Alzheimer's disease (AD) is characterized by brain accumulation of the neurotoxic amyloid-β peptide (Aβ) and by loss of cholinergic neurons and nicotinic acetylcholine receptors (nAChRs). Recent evidence indicates that memory loss and cognitive decline in AD correlate better with the amount of soluble Aβ than with the extent of amyloid plaque deposits in affected brains. Inhibition of nAChRs by soluble Aβ40 is suggested to contribute to early cholinergic dysfunction in AD. Using phage display screening, we have previously identified a heptapeptide, termed IQ, homologous to most nAChR subtypes, binding with nanomolar affinity to soluble Aβ40 and blocking Aβ-induced inhibition of carbamylcholine-induced currents in PC12 cells expressing α7 nAChRs. Using alanine scanning mutagenesis and whole-cell current recording, we have now defined the amino acids in IQ essential for reversal of Aβ40 inhibition of carbamylcholine-induced responses in PC12 cells, mediated by α7 subtypes and other endogenously expressed nAChRs. We further investigated the effects of soluble Aβ, IQ and analogues of IQ on α3β4 nAChRs recombinantly expressed in HEK293 cells. Results show that nanomolar concentrations of soluble Aβ40 potently inhibit the function of α3β4 nAChRs, and that subsequent addition of IQ or its analogues does not reverse this effect. However, co-application of IQ makes the inhibition of α3β4 nAChRs by Aβ40 reversible. These findings indicate that Aβ40 inhibits different subtypes of nAChRs by interacting with specific receptor domains homologous to the IQ peptide, suggesting that IQ may be a lead for novel drugs to block the inhibition of cholinergic function in AD.

## Introduction

Alzheimer's disease (AD) is the most common age-related neurodegenerative disorder and the seventh leading cause of death in the United States [1]. Currently, no effective treatment is available to slow down or stop deterioration of nerve cells in AD. This irreversible disease appears to be initiated by synapse failure, resulting in impairment of cognitive and other cerebral functions [2]. A large body of evidence indicates that the primary agent of neurodegeneration in AD is a 39–43 amino acid residues long peptide known as amyloid-β (Aβ). The majority of secreted Aβ is 40 amino acids long (Aβ40), while the longer, 42-amino acid species (Aβ42) has a high propensity to nucleate and drive the formation of soluble aggregates (e.g., oligomers, protofibrils) and insoluble amyloid fibrils [3], [4], [5]. Substantial evidence indicates that soluble Aβ oligomers are the proximal neurotoxins responsible for synapse dysfunction in AD (for reviews, see [2], [6], [7]. However, the mechanisms linking Aβ40 to synapse dysfunction and neuronal loss remain to be fully elucidated.

A prominent feature of AD pathology is the loss of cholinergic neurons and nicotinic acetylcholine receptors (nAChRs) throughout the brain [8], [9]. With nearly 30 subtypes of brain nAChRs having been described, the three most abundant nAChR subtypes in the mammalian brain are composed of α7, α4β2, and α3β4 subunits [10], expressed in major brain areas including cortex and hippocampus [11]. Although the direct binding of Aβ to α7 receptors has been questioned [12], high-affinity association of Aβ42 with α7 and α4β2 nAChRs has been observed in amyloid plaques and in neurons of AD patients [13], [14], [15], [16]. There is also considerable evidence that Aβ affects the function of nAChRs (for reviews, see [17], [18]). Nanomolar concentrations of Aβ42 or Aβ40 have been reported to inhibit both human and rat homomeric α7 receptors [19], [20], [21], [22], [23], [24]. Moreover, Aβ has been shown to exert subtype-specific actions, activating non-α7 nAChRs in rat basal forebrain neurons [25] and inhibiting non-α7 nAChR subtypes (α4β2, α2β2, α4α5β2) in rodent hippocampal slices [26]. In studies employing heterologously expressed human nAChRs, Aβ has been shown to inhibit α7 and α4β2 subtype function without affecting α3β4 nAChRs [23]. Those effects, however, are still somewhat controversial, as other reports show that picomolar concentrations of Aβ have no effect [23] or even activate whole-cell current responses of α7 nAChRs ([27], [28], [29], [30]; for a review, see [31]).

Using phage-display screening of a peptide library, we previously reported that soluble Aβ binds with nanomolar affinity to a heptapeptide with aminoacid sequence IQTTWSR, henceforth denoted IQ, which is homologous to an amino acid sequence located at the nicotine and acetylcholine (ACh) binding pocket in most subtypes of human nAChRs [24]. Nanomolar concentrations of IQ block Aβ-induced inhibition of carbamylcholine-induced currents in neuronal-differentiated PC12 cells expressing α7 nAChRs, suggesting that inhibition of nAChRs by Aβ results from its binding to the nicotine/ACh binding domain in the receptor. Our previous results further indicated that Aβ interacts with several nAChR subunits homologous to IQ, such as α subunits [24]. Crystallographic studies and alignment of nAChR sequences reveal that the location of the ligand binding site is highly conserved in nAChRs, but the actual ligand binding residues may vary, creating specificities for different ligands [32].

Here, we have used a combination of alanine scanning mutagenesis and rapid kinetic whole-cell current recording [33], [34], [35] to define the amino acid residues in IQ that are essential for alleviating blockade of the inhibition of α7 nAChRs by Aβ40. In addition, we examined the effects of soluble Aβ40, IQ and IQ peptide analogues on α3β4 nAChRs, which are present in human brain but exhibit low homology to α7 in terms of amino acid sequences at the nicotine/ACh binding site. Results show that nanomolar concentrations of soluble Aβ40 inhibit α3β4 nAChRs. In contrast with our previous observations on α7 nAChRs [24], IQ and its analogues do not block Aβ40 inhibition of α3β4 nAChRs. However, simultaneous exposure to IQ and Aβ40 makes the inhibition of α3β4 nAChRs by Aβ40 reversible. These results suggests that Aβ binds to distinct binding sites on different nAChRs subtypes and point to the region homologous to IQ in nAChRs as a relevant target for Aβ40 neurotoxicity in AD.

## Results

### Amino acid residues of IQ involved in blocking inhibition of α7 and other endogenously expressed nAChRs by Aβ40

In order to identify key amino acid residues of the IQ peptide involved in blockade of Aβ-induced inhibition of nAChRs, whole-cell recordings of nAChR currents were carried out in neuronal-differentiated PC12 cells. RT-PCR analysis revealed that such cells express α3, α5, α7, β2 and β4 nAChR subunits, and measurements in the presence of methyllycaconitine (MLA) indicated that, on day 2 following induction to neuronal differentiation, α7 receptors contributed about 50% of cholinergic receptor-mediated whole cell currents [36].

We have previously shown that soluble Aβ40 (200 nM) caused a marked (∼60%) inhibition of nAChR currents and that addition of 500 nM IQ completely blocked this effect. Control measurements showed that addition of IQ alone (up to 500 nM) did not elicit any current in differentiated PC12 cells and (up to 750 nM) did not interfere with currents evoked by carbamylcholine (CCh) [24]. However, at higher concentrations (>1 µM) IQ inhibited nAChR-mediated whole cell currents (I_CCh_) and induced cell death (data not shown), indicating a relatively narrow concentration range in which IQ could be safely used to prevent nAChR inhibition by Aβ40 in the absence of cell toxicity.

We have now investigated a series of IQ analogues for their abilities to block Aβ-induced inhibition of nAChRs in the absence of cell toxicity. A number of peptides were synthesized corresponding to a full alanine scan of the IQ heptapeptide or to truncated tetrapeptides. The impact of those peptides on cell viability was initially tested by the MTT assay, and none of them exhibited cytotoxicity at concentrations of 1 or 100 µM in PC12 cells (Fig. S1). Moreover, no toxic effects exerted by these peptides were observed in HEK cells transfected for α3, β4 receptor expression (data not shown).

**Figure 1:**
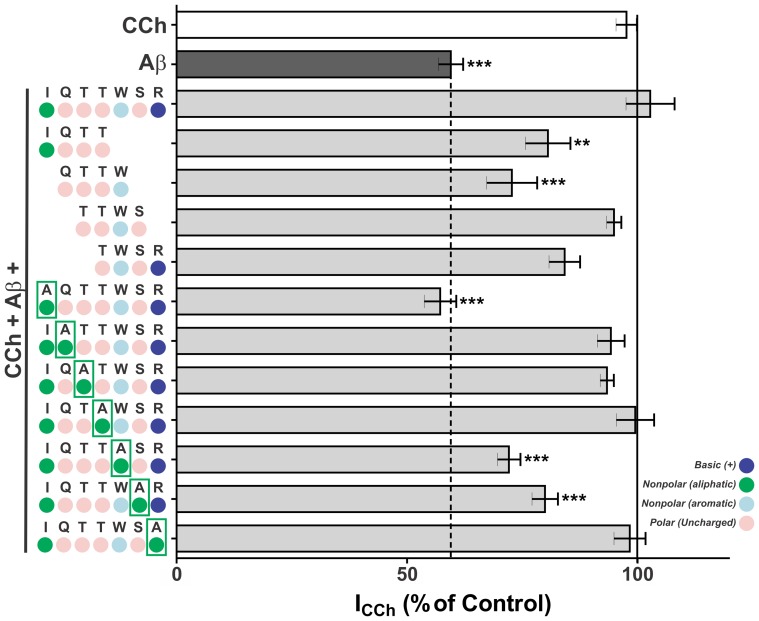
IQ and selected analogues reverse Aβ40 inhibition of nAChRs in PC12 cells. (A) Current responses (normalized by the maximal current evoked by 0.2 mM CCh) of neuronal-differentiated PC12 cells exposed for 2 s to 0.2 mM CCh plus 200 nM Aβ40 in all experimental conditions, except for the control measurement with CCh alone, and, as indicated, 500 nM of different IQ analogues. Bars represent means ± S.D. of at least 3 replicate measurements performed in 4–6 different cells (**, p<0.01; *** p<0.001 in the comparison with the control current evoked by CCh alone).

Each of the IQ analogue peptides (at a fixed concentration of 500 nM, based on our previous results with IQ; ref 24) was then tested for its capacity to alleviate Aβ-induced inhibition of nAChRs in PC12 cells (measured in the presence of 0.2 mM CCh and 200 nM Aβ40 in order to assess maximum inhibition rates; ref 24). Among the tetrapeptides tested, TTWS best mimicked the effect of full-length IQ (Fig. 1), completely reversing Aβ40 inhibition of nAChR-mediated whole cell currents (I_CCh_ 95±2%), followed by TWSR (I_CCh_ 84±4%), IQTT (I_CCh_ 80±5%) and QTTW (I_CCh_ 72±6%). Representative current traces are shown in Fig. S2. Alanine scanning of the IQ sequence showed that replacement of Ile eliminated the capacity to block Aβ-induced inhibition of nAChRs (Ile→Ala, I_CCh_ 57±4%). Moreover, replacement of Trp or Ser residues by Ala resulted in significantly reduced abilities to block Aβ40 inhibition (Trp→Ala, I_CCh_ 72±3%; Ser→Ala, I_CCh_ 79±3%).

### Effects of Aβ, IQ and IQ analogues on α3β4 nAChRs

**Figure 2:**
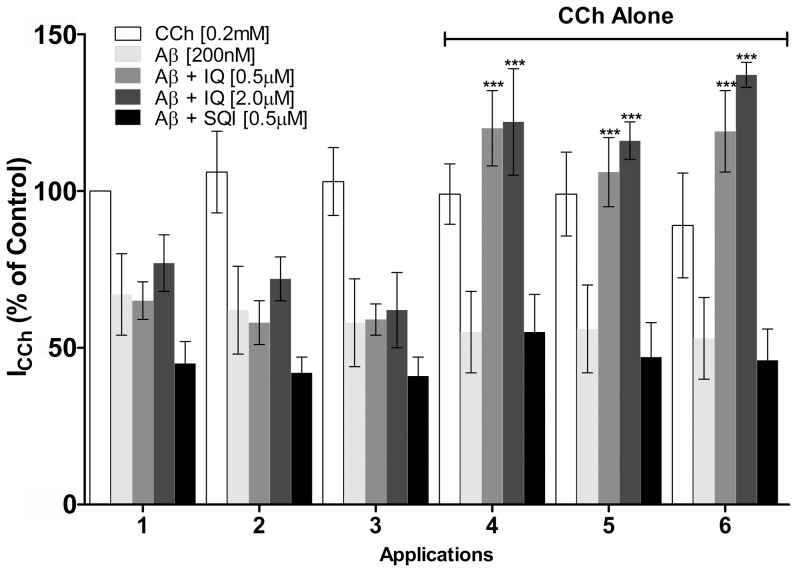
IQ makes Aβ40 inhibition of α3β4 nAChR currents in transformed HEK cells reversible. HEK cells expressing α3β4 nAChRs received consecutive shots (at 5 min intervals) of 0.2 mM CCh plus 200 nM Aβ, in the absence or presence of IQ (0.5 µM) as indicated. Shots 1–3 contained 0.2 mM CCh alone (white bars), 0.2 mM CCh plus 200 nM Aβ (light grey bars), 0.2 mM CCh plus 200 nM Aβ and 0.5/2 µM IQ (grey bars) or 0.5 µM SQI (black bars), used as an inactive control. Shots 4–6 contained 0.2 mM CCh alone for evaluation of reversibility of receptor inhibition. Bars represent mean values ± S.D. of at least 3 replicate measurements (normalized by the maximal current evoked by 0.2 mM CCh) obtained from 4–6 different cells. (***, p<0.001, in comparison with 0.2 mM CCh plus 200 nM Aβ).

Given the abundance of α3β4 receptors in the human brain, we next investigated the inhibition of α3β4 nAChRs by Aβ40 in the presence of 200 nM Aβ40 at effective 0.2 mM CCh concentration. Co-application of 200 nM Aβ40 and 0.2 mM CCh resulted in approximately 35% inhibition of α3β4 nAChR currents in transformed HEK cells (Fig. 2). Successive shots of 0.2 mM CCh on the same cell at 5 min intervals (Fig. 2, white bars, shots 1–6) had no significant effect in the response to CCh, indicating lack of receptor desensitization under these conditions. However, application of three successive shots of 0.2 mM CCh plus 200 nM Aβ40 (Fig. 2, light grey bars, applications 1–3) reduced the cellular response to CCh to approximately 60% of the control level. Subsequent application of three additional shots of 0.2 mM CCh alone to the same cell did not recover the original response to CCh (Fig. 2, light grey bars, shots 4–6), indicating that α3β4 nAChRs remained inhibited even after washout of Aβ. In fact, Aβ-induced inhibition of α3β4 nAChRs persisted even after 6 successive shots of CCh (4 minutes between each shot, comprising approximately 30 minutes for each experiment) following a single initial exposure to 0.2 mM CCh plus 200 nM Aβ40 (Fig. S3).

Irreversible inhibition of α3β4 receptors by Aβ was also observed following three shots of 0.2 mM CCh plus 200 nM Aβ40 and 0.5 µM SQI (a control scrambled peptide that has the same amino acid composition as IQ but does not bind to Aβ), followed by three shots of 0.2 mM CCh alone (Fig. 2, black bars). Cells that had been exposed to three shots of 0.2 mM CCh plus 200 nM Aβ40 in the presence of 0.5 or 2 µM IQ presented reduced response to CCh stimulation (∼60% and 70% of control currents, respectively; Fig. 2, grey bars, shots 1–3). Thus, in contrast with its ability to block inhibition of α7 nAChRs [24], IQ was not capable of preventing the inhibition of α3β4 nAChRs by Aβ. Interestingly, however, the response of α3β4 receptors to CCh (Fig. 2, grey bars, shots 4–6) returned to control levels during the washout period after the co-application of CCh, Aβ40 and IQ. This indicates that, in the presence of IQ, the inhibition of α3β4 receptors by Aβ40 becomes reversible following Aβ40 washout.

As a control, we tested whether IQ, QI (a peptide with a reverse sequence compared to IQ) or SQI induced activation of α3β4 nAChR currents or had any impact on cellular response to CCh. Results showed that none of the three peptides by themselves elicited currents or had any detectable effect on whole-cell current responses of PC12 cells (Fig. S4), supporting the notion that rescue of cellular α3β4 nAChR response to CCh by IQ is due to its interaction with Aβ.

**Figure 3:**
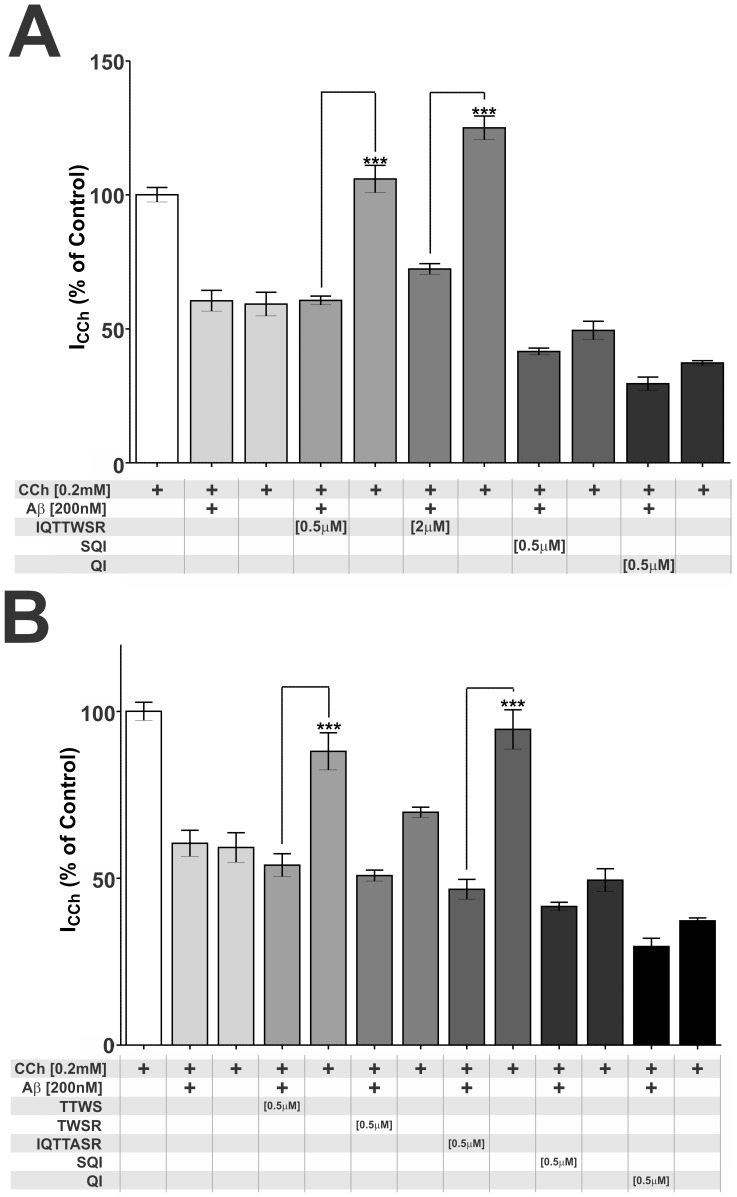
Effects of IQ and analogues on Aβ40 inhibition of α3β4 nAChRs in transformed HEK cells. (A) HEK cells expressing recombinant α3β4 nAChRs received 3 consecutive shots (at 4 min intervals) of 0.2 mM CCh plus 200 nM Aβ40 in the absence or presence of 0.5 and 2 µM IQTTWSR. QI and SQI (500 nM) were used as ineffective control peptides. Recovery of current response was evaluated after washout and 3 shots of CCh alone. (B) HEK cells expressing recombinant α3β4 nAChRs received 3 consecutive shots of 0.2 mM CCh plus 200 nM Aβ40 in the absence or presence of 500 nM TTWS, TWSR or IQTTASR. QI and SQI (500 nM) were used as ineffective control peptides. Recovery of current response was evaluated after washout and 3 shots of CCh alone. Bars represent mean values ± S.D. of current responses (normalized by the maximal current evoked by 0.2 mM CCh) of at least 3 measurements performed in at least 3 different cells. (***, p<0.001).

Finally, we evaluated the effects of IQ and selected peptide analogues on the inhibition of α3β4 nAChRs by Aβ. To this end, cells received three shots of each peptide as shown in Fig. 3. For all cells analyzed (at least 3 cells per experimental condition), currents measured in the presence of the peptides were compared to those measured in the presence of CCh alone or CCh+Aβ. For each cell, 3 shots (with a 4 minute interval between them) of CCh were applied to elicit maximum responses, then 3 shots of CCh+Aβ to induce inhibition, followed by 3 shots of CCh+Aβ+peptide, and finally 3 more shots of CCh alone in order to verify the persistence of inhibition.

In the absence of peptides, inhibition by Aβ40 was found to be persistent when CCh alone was applied after the shots of CCh+Aβ. Interestingly, when shots included CCh+Aβ+IQTTWSR (0.5 or 2 µM), α3β4 nAChR currents were rescued from inhibition when measured in the presence of CCh alone (after washout of Aβ). We next tested the effects of the TTWS and TWSR tetrapeptides, which had shown the best protective actions against Aβ-induced inhibition of nAChRs, and IQTTASR, which lacks the highly conserved Trp residue in the agonist-binding domain of nAChRs and presented the lowest capacity to alleviate Aβ40 inhibition of α7 currents (Fig. 1). A slight increase in Aβ-induced inhibition was observed in the presence of SQI, which, however, was not statistically significant.

When tested on α3β4 nAChR-expressing cells, all peptides tested failed in preventing the inhibition of α3β4 nAChR-mediated currents by Aβ. However, when Aβ40 was added to cells in conjunction with IQ, TTWS or IQTTASR (0.5 µM each), α3β4 nAChR-mediated currents in response to CCh alone returned to approximately 100%, 88% and 94%, respectively, of control levels. On the other hand, TWSR, QI and SQI peptides were unable to rescue the inhibition of CCh-induced α3β4 nAChR-mediated responses by Aβ40 (Fig. 3).

## Discussion

We have previously identified an Aβ40 ligand, a peptide termed IQ, that blocks Aβ-induced inhibition of nAChRs at nanomolar concentrations [24]. IQ is homologous to the ligand-binding domain of nAChRs. The location of the ligand-binding site is conserved among different pentameric ligand gated ion channel receptors, but the actual ligand binding residues may vary, creating specificities for different ligands [32]. Therefore, we proposed that Aβ40 might interact with the ligand-binding domain of distinct nAChRs subunits, preferentially binding to those with higher homology to IQ, such as α7. This is consistent with previous reports of higher affinity interactions between Aβ40 and α7 than with α4β2 nAChRs from rat and guinea pig cerebral cortex and from hippocampal synaptic membranes [13], [14]. Neuronal nAChRs are assembled as homomeric or heteromeric combinations of α (α2–10) and β (β2–4) subunits. The majority of human CNS nAChRs is of the α4β2 subtype and the remainder is largely made up of α7 subunit homopentamers and α3β4 heteromers, although several other combinations are also known [10], [23].

Here, we have asked which amino acid residues of IQ are essential for blockade of Aβ40 inhibition of receptor currents in cells containing α7 or heteromeric nicotinic receptors, and specifically tested the effects of soluble Aβ, IQ and IQ analogues in cells expressing only the α3β4 nAChR subtype. We used a whole-cell current-recording approach in combination with the cell-flow technique [33] to briefly expose differentiated PC12 cells or HEK cells expressing α3β4 nAChRs to Aβ40 and other ligands. This procedure minimizes receptor desensitization and avoids long periods of incubation with Aβ, assuring preservation of Aβ40 in soluble state during the experiments, as previous described [24].

Co-application of 0.2 mM CCh, 200 nM Aβ40 and 500 nM of different IQ analogues to differentiated PC12 cells showed that, among the tetrapeptides tested, TTWS was the analogue that best emulated the protective effect of full-length IQ, completely preventing Aβ-induced inhibition of nAChRs (I_CCh_ 95±2%). Next in terms of effectiveness were TWSR, IQTT and QTTW. Both TTWS and TWSR contain Trp57, a highly conserved residue present in the sequences of all nAChRs described so far [37]. Trp57 has been shown to be important for binding of d-tubocurarine (a competitive antagonist of nAChRs) to *Torpedo* nAChR [38]. Both peptides also contain a Ser residue (Ser58) present in 1 of the 12 human nAChR subunit sequences and conservatively replaced by Thr in 5 of the remainder 11 sequences. Ala-scanning of the IQ sequence indicated that the Trp and Ser residues of IQ are essential for efficacy in preventing Aβ40 inhibition of nicotinic receptors. Ala substitutions also pointed to the importance of Ile in the IQ sequence. Significantly, Ile53 (or its highly conserved substitution Leu) is present in 11 of the 12 human nAChR subunits known to date.

**Figure 4:**
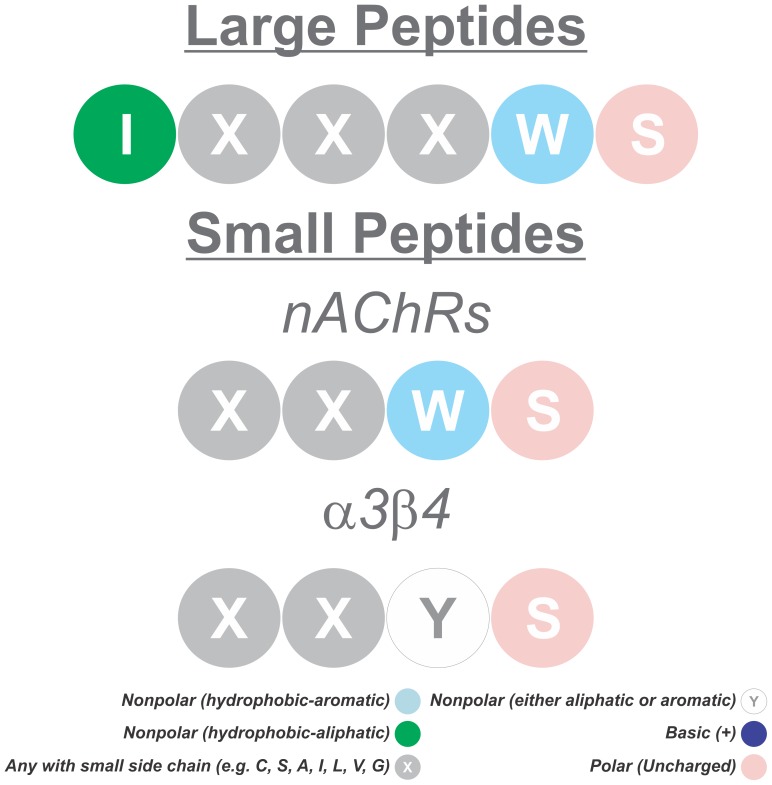
Suggested conserved amino acid sequence for reversal of α3β4 nAChR inhibition by Aβ.

On the other hand, replacement of Gln, Thr or Arg residues by Ala did not significantly affect the efficacy of IQ analogues (Fig. 1), despite the fact that mutations in Gln56 (numbering according to the α7 nAChR sequence) affect the affinities for ACh and nicotine [39]. Collectively, these results show that Ile, Trp and Ser residues in the amino acid sequence of IQ (IQTTWSR) are essential to block Aβ40 inhibition of nAChRs. Based on these findings, we propose that protection by longer peptides (containing 6 amino acid residues or more) can be explained on the basis of a sequence motif in which Ile, Trp and Ser residues at positions 1, 5 and 6, respectively, are conserved (i.e., IxxxWS). A similar model can be developed for shorter peptides (of 4 amino acid residues or less) and also for protection against inhibition of α3β4 receptors and possibly other nicotinic subtypes by Aβ40 (Fig. 4). For α7 nicotinic receptors and other subtypes expressed by PC12 cells, carboxyterminal Trp and Ser residues must be conserved to preserve efficacy of tetrapetides in blocking Aβ40 inhibition. On the other hand, our results show that for α3β4 receptors the Trp residue can be replaced by a nonpolar (aliphatic or aromatic) amino acid residue without loss in activity (Fig. 3B). Defining these structural motifs may prove useful for development of novel IQ analogues with improved efficacy in protection against Aβ40 inhibition of nAChRs and/or recovery from such inhibition, and as a molecular backbone for development of non-peptide drugs.

The fact that IQ is homologous to several nAChR subunits [24] suggests that Aβ40 binds to this highly conserved domain in different nAChRs subtypes. Although direct binding was not tested in the present study, we assume a similar mechanism of action for the other tested peptides. In order to test this hypothesis, we tested the effects of Aβ, IQ and IQ analogues in a cell line expressing a single subtype of nAChR, the α3β4 subtype, characterized by large whole-cell current responses and widely used as model for binding and activity screening on nicotinic receptors [40], [41], [42], [43], [44]. Moreover, this receptor subtype was recently employed to characterize the mechanism of action of the Alzheimer drug tacrine [42] and has also been shown to be involved in disease states such as nicotine-induced seizures and hypolocomotion in mice [45]. Results showed that 200 nM soluble Aβ40 persistently blocked the response of α3β4 nAChRs to CCh remaining 60±14% of currents induced by CCh alone. To our knowledge, there is only one other study testing the effects of Aβ40 on α3β4 nAChRs [23]. That study showed that Aβ failed to elicit changes in amplitude of ACh-evoked currents mediated by human α3β4 nAChRs expressed in *Xenopus laevis* oocytes. It should be noted, however, that Aβ40 was bath applied at a significantly lower concentration (10 nM) than used in the present study (200 nM). Moreover, Pym et al. [23] pre-incubated Aβ40 with cells for 3 min, which might lead to aggregation and, consequently, to a decrease in the concentration of soluble Aβ40 species that directly interact with nAChRs. Although the concentration of Aβ40 in the cerebrospinal fluid of AD patients has been reported to be between 1 and 10 nM [46], the concentration of Aβ40 at cholinergic synapses is unknown.

In contrast to their effects in cells expressing α7 and heteromeric nAChRs, IQ and analogues did not block Aβ40 inhibition of α3β4 nAChRs expressed in HEK cells, suggesting that IQ binding to Aβ40 is not sufficient to prevent Aβ from interacting with and inhibiting α3β4 receptors. However, addition of IQ made the inhibition of α3β4 nAChRs by Aβ40 reversible, suggesting that IQ binding to Aβ40 modifies its interaction with α3β4 receptors, likely facilitating Aβ40 dissociation and receptor re-activation by the agonist. Activity screening of IQ analogues indicated that only TTWS and IQTTASR were able to mimic IQ and make Aβ40 inhibition of α3β4 nAChRs reversible, emphasizing the importance of the TTWS tetrapeptide in Aβ40 interaction with nAChRs. Unexpectedly, the Trp residue (present in all nAChRs) that is essential in IQ to block Aβ40 inhibition of α7 nAChRs was not necessary to alleviate the effects of Aβ40 on α3β4 nAChRs, suggesting that different amino acid residues or different protein domains are involved in Aβ40 interaction with distinct subtypes of nAChRs.

Current results support the notion that Aβ40 binds with distinct affinities to and has different effects on various subtypes of nAChRs [6], [27]. Indeed, it has been reported that Aβ binds with high affinity (in the picomolar range) to α7 nAChRs in cortical regions and in the hippocampus in AD, and with about 5,000 times lower affinity to α4β2 nAChRs [13], [14]. However, as a general mechanism, blockade of nAChRs by Aβ may also affect, at concentrations similar to those used in the present study, the cholinergic control of neurotransmitter release, including glycine, glutamate, aspartate and GABA [47], [48].

Distinct effects of Aβ on different subtypes of nAChRs reinforce the idea that Aβ binding to different receptor subtypes may involve different binding sites, occasionally increasing but more often blocking agonist response. Moreover, the difference in effects of IQ and analogues in cells expressing α7 and other heteromeric receptors versus in cells solely expressing α3β4 nAChRs may be due to the fact that IQ presents higher homology to the ligand binding pocket of α7 than of α3β4 nAChRs. In nAChRs, the ligand-binding site is located at the interface between two subunits [46], [49]. Numerous biochemical studies have shown that the principal part of the binding site is formed by α-subunit residues [50], [51], [52], [53], whereas neighboring subunit residues contribute to form the complementary part of the binding pocket. Thus, heteropentamers such as α3β4 subtype contain two different ligand-binding sites with distinct affinities, whereas the homopentameric α7 receptor contains five identical ligand-binding sites [32]. The most vulnerable neurons in AD seem to be those expressing high levels of nAChRs, particularly those containing the α7 subunit [54], and levels of nAChRs as well as some of their associated proteins decrease in AD [55], [56]. An interesting recent study reported that deletion of the α7 nAChR gene prevents cognitive deficits and synaptic pathology in a mouse model of Alzheimer's disease [57]. Our current results provide novel information to drive further progress in AD drug design. Drugs like IQ, capable of disrupting Aβ-α7 nAChR interactions, might alleviate Aβ-mediated toxicity and block AD development.

In conclusion, our finding that Aβ exerts subtype-specific inhibitory effects on α7 and α3β4 nAChRs suggests that receptor subunit composition might account for some of the different actions reported for Aβ40 on neurons *in vivo*. Furthermore, we show that the region homologous to IQ in nAChRs is a relevant target to alleviate blockade of α7 and α3β4 nAChRs by Aβ. Our results identify, for the first time, the amino acid residues probably involved in binding and inhibition of nAChRs by Aβ and may provide a valuable platform for drug design of novel AD therapeutics. The potential relevance of our findings to drug design and development of novel AD treatments is further underscored by a recent string of disappointing clinical trials on Aβ antibodies (Bapineuzumab and Solanezumab), which have cast a shadow over anti-Aβ immunotherapy strategies [58].

## Materials and Methods

### Peptide synthesis

Peptides IQ (IQTTWSR), AQTTWSR, IATTWSR, IQATWSR, IQTAWSR, IQTTASR, IQTTWAR, IQTTWSA, IQTT, QTTW, TTWS, TWSR and scrambled IQ (SQI;.TIWQSTR) were synthesized as detailed elsewhere [24].

### Cell culture

PC12 cells (ATCC, catalogue # CRL-1721) were cultured and induced to neuronal differentiation as described [24], [36]. Briefly, PC12 cells were cultured in DMEM (Invitrogen, Life Technologies, Carlsbad, CA, USA) in the presence of 10% FBS (Cultilab, Campinas, São Paulo, Brazil), 5% horse serum (Invitrogen, Life Technologies, Carlsbad, CA, USA), streptomycin (100 µg/ml), penicillin (100 U/ml – Sigma-Aldrich, St. Louis, MO, USA) and 1 mM sodium pyruvate (Invitrogen, Life Technologies, Carlsbad, CA, USA). N^5^,2′-O-dibutyryl cAMP (dibutyril cAMP) and FGF-2 (45 ng/ml) were added to cultures to induce differentiation into mature sympathetic neurons expressing increased numbers of neuronal nAChRs [59]. For differentiation, 2.5×10^5^ cells/ml, as determined by Neubauer chamber counting, were induced to neuronal differentiation for up to 6 days in DMEM containing 30 ng/ml FGF-2 (Sigma-Aldrich, St. Louis, MO, USA) and 250 µM dibutyril cAMP (Sigma-Aldrich, St. Louis, MO, USA). Under these conditions, differentiated PC12 cells express α3, α5, α7, β2 and β4 nicotinic receptor subunits [36]. For evaluation of cell viability, PC12 cells on day 3 of neuronal differentiation were exposed for 48 h to different peptides at 1 and 100 µM concentrations, then washed with PBS and stained with trypan blue. Five fields were photographed per well and live and dead cells were counted. Statistical analysis was based on the Student's t-test.

Human embryonic kidney cells (HEK293 cells) stably expressing rat α3 and β4 nAChR subunits [60] were obtained from Dr. Yingxian Xiao, Georgetown University. Transfected cells were cultured in DMEM (Invitrogen, Life Technologies, Carlsbad, CA, USA) supplemented with 10% FBS, 100 U/ml penicillin G, 100 µg/ml streptomycin and 0.3 mg/ml geneticine (Sigma-Aldrich, St. Louis, MO, USA) at 37°C and 5% CO_2_. Cells were allowed to attach to 35 mm cell culture dishes for 48 h prior to being used in whole-cell recording experiments.

### Whole-cell current recording and rapid application of ligand solutions (cell flow technique)

PC12 cells following 3–6 days of neuronal differentiation were cultured at a density of 20–100 cells/mm^2^ on 35 mm cell culture dishes. Whole-cell recordings were performed at room temperature at a transmembrane voltage of −70 mV. The solution in the recording pipette contained 145 mM KCl, 10 mM NaCl, 2 mM MgCl_2_, 1 mM EGTA, 25 mM HEPES, pH 7.4. The bath solution was composed of 145 mM NaCl, 5.3 mM KCl, 1.8 mM CaCl_2_, 1.2 mM MgCl_2_, 10 mM glucose, 25 mM HEPES, pH 7.4. Further details were previously reported [24].

Using carbamylcholine (CCh), a stable analog of ACh, we have previously shown that whole-cell current (I*_CCh_*) data in neuronal-differentiated PC12 cells could be well described by a single binding site model, yielding a K_d_ of 259±58 µM for CCh. A K_d_ value of 2 mM has already been determined for α3β4 nAchRs expressed by HEK cells [61]. According to previous work published by Niu et al., 1995 and Hess et al. 2000 [62], [63], the equilibrium between open and closed channel forms is defined by the concentration of the agonist, thereby the closed channel form reveals higher affinity for the inhibitor as the open channel form does. Therefore, higher percentages of inhibition by Aβ40 are expected at low CCh (0.2 mM) concentration. Because the density of receptors in the plasmamembrane (i.e., the total number of binding sites) differs somewhat from cell to cell, all I_CCh_ values were normalized to the currents measured in the presence of 0.2 mM CCh [24]. CCh-induced currents were recorded by whole-cell recording in combination with a rapid kinetic ligand delivery system, denominated the cell-flow technique, which provides a time resolution of 10 ms [24], [33], [34], [35]. Briefly, a U-shaped stainless steel capillary tube (250 µm i.d.) with a circular porthole of 150 µm in diameter at the base of the U was connected to pumps on both ends so the solution containing ligand could be driven into the tube at one end and removed through the other end at twice the entry flow rate [33]. The porthole was placed about100 µm away from each cell clamped by the recording pipette. Upon closing a solenoid valve between the U-tube and the suction pump by an electric trigger, CCh, Aβ40 and/or different peptides were applied to the cell in a laminar flow. Aβ40 and peptide solutions were mixed prior to co-application with CCh. Recorded signals were amplified using an Axopatch 200B amplifier (Molecular Devices, LLC, Sunnyvale, CA, USA) and filtered at 2 KHz using a 40-pole low-pass Bessel filter. The filtered signals were digitized using a Digidata 1322A interface, recorded using the pCLAMP software package (Molecular Devices) and analyzed using Microcal Origin software (Microcal Software, Inc., North Hampton, MA, USA). Statistical analysis was performed by comparing mean values using one-way analysis of variance (ANOVA) with Bonferroni's correction.

### Correction for receptor desensitization in cell-flow measurements

The maximum current amplitude is proportional to the density of open channels. As receptor desensitization may occur while the ligand solution is equilibrating with the cell surface, observed current amplitudes are corrected for desensitization using the equation [33], [35]:
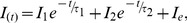
(1)where I(t) is the maximum current amplitude at time t; I_1_, I_2_, I_e_ are the maximum current amplitudes for the first, second, and equilibrium current decay components, respectively; and τ_1_ and τ_2_ are the time constants for the first and second components (fast and slow receptor desensitization, respectively). Origin software (Microcal Software, Inc.) was used to estimate the rate of current decay in the presence of agonist. Equation 1 was fitted to the decreasing part of the recording and the observed maximum current amplitude was corrected for receptor desensitization accordingly [33].

## Supporting Information

Figure S1 **Cytotoxicity assay selected IQ analogues.** PC12 cells induced to neuronal differentiation were incubated in the presence of different peptides for 48 hours, washed with PBS and stained with trypan blue. The percentages of live and dead cells of five fields per well were counted and compared to those of control cells incubated in the absence of peptides.
(TIFF)

Figure S2 **Current traces of different peptides tested for reversion of α3β4 nAChR inhibition by Aβ40.** Current responses (normalized by the maximal current evoked by 0.2 mM CCh) of neuronal differentiated PC12 cells exposed for 2 s to 0.2 mM CCh plus 200 nM Aβ40 in all experimental conditions, except for the control measurement with CCh alone, and, as indicated, 500 nM of different IQ analogues. The here shown original data are illustrative for mean values ± S.D. reported in Fig. 1.
(TIF)

Figure S3 **Aβ40-induced inhibition of α3β4 nAChR currents in transformed HEK cells persists after washout.** Following six consecutive applications of 0.2 mM CCh, 0.2 mM CCh was co-applied once in the presence of 200 nM Aβ40. Following washout of Aβ, inhibition persisted in six consecutive applications of CCh (p<0.005, when compared to control currents measured prior to Aβ administration).
(TIF)

Figure S4 **IQ, QI or SQI alone do not instigate nAChR currents in PC12 cells.** The initial whole-cell response induced by 0.2 mM CCh was normalized to 100% of activity. None of the peptides (IQ, QI, SQI, tested at 2 µM) induced changes in CCh-evoked currents nor activated receptor responses in the absence of agonist. Arrows indicate time points of ligand application.
(TIF)

## References

[1] Alzheimer's Association (2010) Alzheimer's disease facts and figures. Alzheimers Dement 6: 158–194. 20298981 10.1016/j.jalz.2010.01.009

[2] Ferreira ST, Klein WL (2011) The Aβ oligomer hypothesis for synapse failure and memory loss in Alzheimer's disease. Neurobiol Learn Mem 96: 529–543. 21914486 10.1016/j.nlm.2011.08.003 PMC4390395

[3] Lambert MP, Barlow AK, Chromy BA, Edwards C, Freed R, et al (1998) Diffusible, nonfibrillar ligands derived from Abeta1-42 are potent central nervous system neurotoxins. Proc Natl Acad Sci USA 95: 6448–6453. 9600986 10.1073/pnas.95.11.6448 PMC27787

[4] Riek R (2006) Cell biology: infectious Alzheimer's disease? Nature 444: 429–431. 17122841 10.1038/444429a

[5] Greenwald J, Riek R (2010) Biology of amyloid: structure, function, and regulation. Structure 18: 1244–1260. 20947013 10.1016/j.str.2010.08.009

[6] Ferreira ST, Vieira MN, De Felice FG (2007) Soluble protein oligomers as emerging toxins in Alzheimer's and other amyloid diseases. IUBMB Life 59: 332–345. 17505973 10.1080/15216540701283882

[7] Selkoe DJ (2008) Soluble oligomers of the amyloid beta-protein impair synaptic plasticity and behavior. Behav Brain Res 192: 106–113. 18359102 10.1016/j.bbr.2008.02.016 PMC2601528

[8] Nordberg A (2001) Nicotinic receptor abnormalities of Alzheimer's disease: therapeutic implications. Biol Psychiatry 49: 200–210. 11230871 10.1016/s0006-3223(00)01125-2

[9] Kadir A, Almkvist O, Wall A, Långström B, Nordberg A (2006) PET imaging of cortical 11C-nicotine binding correlates with the cognitive function of attention in Alzheimer's disease. Psychopharmacology (Berl) 188: 509–520. 16832659 10.1007/s00213-006-0447-7

[10] Lindstrom JM (2003) Nicotinic acetylcholine receptors of muscles and nerves: comparison of their structures, functional roles, and vulnerability to pathology. Ann N Y Acad Sci 998: 41–52. 14592862 10.1196/annals.1254.007

[11] Clementi F, Fornasari D, Gotti C (2000) Neuronal nicotinic receptors, important new players in brain function. Eur J Pharmacol 393: 3–10. 10770992 10.1016/s0014-2999(00)00066-2

[12] Small DH, Maksel D, Kerr ML, Ng J, Hou X, et al (2007) The beta-amyloid protein of Alzheimer's disease binds to membrane lipids but does not bind to the α7 nicotinic acetylcholine receptor,. J Neurochem 101: 1527–1538. 17286584 10.1111/j.1471-4159.2006.04444.x

[13] Wang HY, Lee DH, D'Andrea MR, Peterson PA, Shank RP, et al (2000) Beta-Amyloid (1-42) binds to alpha7 nicotinic acetylcholine receptor with high affinity. Implications for Alzheimer's disease pathology. J Biol Chem 25: 5626–5632. 10.1074/jbc.275.8.5626 10681545

[14] Wang HY, Lee DH, Davis CB, Shank RP (2000) Amyloid peptide Aβ1–42 binds selectively and with picomolar affinity to α7 nicotinic acetylcholine receptors. J Neurochem 75: 1155–1161. 10936198 10.1046/j.1471-4159.2000.0751155.x

[15] Nagele RG, D'Andrea MR, Anderson WJ, Wang HY (2002) Intracellular accumulation of beta-amyloid (1-42) in neurons is facilitated by the alpha 7 nicotinic acetylcholine receptor in Alzheimer's disease. Neurosci 110: 199–211. 10.1016/s0306-4522(01)00460-2 11958863

[16] Wang HY, Bakshi K, Shen C, Frankfurt M, Trocmé-Thibierge C, et al (2010) S 24795 limits beta-amyloid-alpha7 nicotinic receptor interaction and reduces Alzheimer's disease-like pathologies. Biol. Psychiatry 67: 522–530. 10.1016/j.biopsych.2009.09.031 19932469

[17] Dineley KT (2007) Beta-amyloid peptide-nicotinic acetylcholine receptor interaction: the two faces of health and disease. Front Biosci 12: 5030–5038. 17569627 10.2741/2445

[18] Jürgensen S, Ferreira ST (2010) Nicotinic receptors, amyloid-beta, and synaptic failure in Alzheimer's disease. J Mol Neurosci 40: 221–229. 19690986 10.1007/s12031-009-9237-0

[19] Pettit DL, Shao Z, Yakel JL (2001) Beta-Amyloid1–42 peptide directly modulates nicotinic receptors in the rat hippocampal slice. J Neurosci 21: RC120. 11150356 10.1523/JNEUROSCI.21-01-j0003.2001 PMC6762461

[20] Liu QS, Kawai H, Berg DK (2001) Beta-amyloid peptide blocks the response of alpha 7-containing nicotinic receptors on hippocampal neurons. Proc Natl Acad Sci USA 98: 4734–9. 11274373 10.1073/pnas.081553598 PMC31903

[21] Grassi F, Palma E, Tonini R, Amici M, Ballivet V, et al (2003) Amyloid beta (1-42) peptide alters the gating of human and mouse alpha-bungarotoxin-sensitive nicotinic receptors. J Physiol 547: 147–157. 12562926 10.1113/jphysiol.2002.035436 PMC2342606

[22] Wu J, Kuo YP, George AA, Xu L, Hu J, et al (2004) beta-Amyloid directly inhibits human alpha4beta2-nicotinic acetylcholine receptors heterologously expressed in human SH-EP1 cells. J Biol Chem 279: 37842–37851. 15234980 10.1074/jbc.M400335200

[23] Pym L, Kemp M, Raymond-Delpech V, Buckingham S, Boyd CA, et al (2005) Subtype-specific actions of beta-amyloid peptides on recombinant human neuronal nicotinic acetylcholine receptors (a7, a4b2, b3b4) expressed in Xenopus laevis oocytes,. Br J Pharmacol 146: 964–971. 16184187 10.1038/sj.bjp.0706403 PMC1751230

[24] Magdesian MH, Nery AA, Martins AH, Juliano MA, Juliano L, et al (2005) Peptide blockers of the inhibition of neuronal nicotinic acetylcholine receptors by amyloid beta. J Biol Chem 280: 31085–31090. 15987688 10.1074/jbc.M502406200

[25] Fu W, Jhamandas JH (2003) Beta-amyloid peptide activates non-alpha7 nicotinic acetylcholine receptors in rat basal forebrain neurons. J Neurophysiol 90: 3130–3136. 12890800 10.1152/jn.00616.2003

[26] Lamb PW, Melton MA, Yakel JL (2005) Inhibition of neuronal nicotinic acetylcholine receptor channels expressed in Xenopus oocytes by betaamyloid1–42 peptide. J Mol Neurosci 27: 13–21. 16055943 10.1385/JMN:27:1:013

[27] Dineley KT, Bell KA, Bui D, Sweatt JD (2002) Beta-Amyloid peptide activates alpha 7 nicotinic acetylcholine receptors expressed in Xenopus oocytes. J Biol Chem 277: 25056–25061. 11983690 10.1074/jbc.M200066200

[28] Dougherty JJ, Wu J, Nichols RA (2003) Beta-amyloid regulation of presynaptic nicotinic receptors in rat hippocampus and neocortex. J Neurosci 23: 6740–6747. 12890766 10.1523/JNEUROSCI.23-17-06740.2003 PMC6740736

[29] Wu J, Khan GM, Nichols RA (2007) Dopamine release in prefrontal cortex in response to beta-amyloid activation of alpha7 nicotinic receptors. Brain Res 1182: 82–89. 17935702 10.1016/j.brainres.2007.08.079 PMC2153437

[30] Puzzo D, Privitera L, Leznik E, Fà M, Staniszewski A, et al (2008) Picomolar amyloid-beta positively modulates synaptic plasticity and memory in hippocampus. J Neurosci 28: 14537–14545. 19118188 10.1523/JNEUROSCI.2692-08.2008 PMC2673049

[31] Buckingham SD, Jones AK, Brown LA, Sattelle DB (2009) Nicotinic acetylcholine receptor signalling: roles in Alzheimer's disease and amyloid neuroprotection. Pharmacol Rev 61: 39–61. 19293145 10.1124/pr.108.000562 PMC2830120

[32] Brejc K, van Dijk WJ, Klaassen RV, Schuurmans M, van Der Oost J, et al (2001) Crystal structure of an ACh-binding protein reveals the ligand-binding domain of nicotinic receptors. Nature 411: 269–276. 11357122 10.1038/35077011

[33] Udgaonkar JB, Hess GP (1987) Chemical kinetic measurements of a mammalian acetylcholine receptor by a fast-reaction technique. Proc Natl Acad Sci U S A 84: 8758–8762. 2447583 10.1073/pnas.84.24.8758 PMC299629

[34] Ulrich H, Ippolito JE, Pagán OR, Eterović VA, Hann RM, et al (1998) In vitro selection of RNA molecules that displace cocaine from the membrane-bound nicotinic acetylcholine receptor. Proc Natl Acad Sci U S A 95: 14051–14056. 9826651 10.1073/pnas.95.24.14051 PMC24324

[35] Ulrich H, Akk G, Nery AA, Trujillo CA, Rodriguez AD, et al (2008) Mode of cembranoid action on embryonic muscle acetylcholine receptor. J Neurosci Res 86: 93–107. 17868151 10.1002/jnr.21468

[36] Nery AA, Resende RR, Martins AH, Trujillo CA, Eterovic VA, et al (2010) Alpha 7 nicotinic acetylcholine receptor expression and activity during neuronal differentiation of PC12 pheochromocytoma cells. J Mol Neurosci 4: 329–339. 10.1007/s12031-010-9369-2 20461497

[37] Williams DK, Stokes C, Horenstein NA, Papke RL (2009) Differential regulation of receptor activation and agonist selectivity by highly conserved tryptophans in the nicotinic acetylcholine receptor binding site. J Pharmacol Exp Ther 330: 40–53. 19339660 10.1124/jpet.109.151225 PMC2700159

[38] Chiara DC, Cohen JB (1997) Identification of amino acids contributing to high and low affinity d-tubocurarine sites in the Torpedo nicotinic acetylcholine receptor. J Biol Chem 272: 32940–32950. 9407073 10.1074/jbc.272.52.32940

[39] Corringer PJ, Galzi JL, Eisele JL, Bertrand S, Changeux JP, et al (1995) Identification of a new component of the agonist binding site of the nicotinic alpha 7 homooligomeric receptor. J Biol Chem 270: 11749–11752. 7744821 10.1074/jbc.270.20.11749

[40] Cheffer A, Mustafá EV, T-do Amaral A, Ulrich H (2012) Lipophilicity as a determinant of binding of procaine analogs to rat α3β4 nicotinic acetylcholine receptor. J Neurosci Res 90: 1607–1614. 22504865 10.1002/jnr.23047

[41] Moaddel R, Jozwiak K, Yamaguchi R, Cobello C, Whittington K, et al (2004) On-line screening of conformationally constrained nicotines and anabasines for agonist activity at the alpha3beta4- and alpha4beta2-nicotinic acetylcholine receptors using immobilized receptor-based liquid chromatographic stationary phases. J Chromatogr B Analyt Technol Biomed Life Sci 813: 235–240. 10.1016/j.jchromb.2004.09.042 15556538

[42] Cheffer A, Ulrich H (2011) Inhibition mechanism of rat α3β4 nicotinic acetylcholine receptor by the Alzheimer therapeutic tacrine. Biochemistry 50: 763–770. 21247200 10.1021/bi101789y

[43] Nunes-Alves A, Nery AA, Ulrich H (2013) Tobacco nitrosamine N-nitrosonornicotine as inhibitor of neuronal nicotinic acetylcholine receptors. J Mol Neurosci 49: 52–61. 22847530 10.1007/s12031-012-9859-5

[44] Azam L, McIntosh JM (2009) Alpha-conotoxins as pharmacological probes of nicotinic acetylcholine receptors. Acta Pharmacol Sin 30: 771–783. 19448650 10.1038/aps.2009.47 PMC2814007

[45] Salas R, Cook KD, Bassetto L, De Biasi M (2004) The alpha3 and beta4 nicotinic acetylcholine receptor subunits are necessary for nicotine-induced seizures and hypolocomotion in mice. Neuropharmacology 47: 401–417. 15275829 10.1016/j.neuropharm.2004.05.002

[46] Mehta PD, Pirttila T, Patrick BA, Barshatzky M, Mehta SP (2001) Amyloid beta protein 1-40 and 1-42 levels in matched cerebrospinal fluid and plasma from patients with Alzheimer disease. Neurosci Lett 304: 102–106. 11335065 10.1016/s0304-3940(01)01754-2

[47] Zappettini S, Grilli M, Olivero G, Mura E, Preda S, et al (2012) Beta amyloid differently modulate nicotinic and muscarinic receptor subtypes which stimulate in vitro and in vivo the release of Glycine in the rat hippocampus. Front Pharmacol 3: 146. 22866037 10.3389/fphar.2012.00146 PMC3406330

[48] Mura E, Zappettini S, Preda S, Biundo F, Lanni C, et al (2012) Dual effect of beta-amyloid on α7 and α4β2 nicotinic receptors controlling the release of glutamate, aspartate and GABA in rat hippocampus. PLoS One 2012;7 (1) e29661. 10.1371/journal.pone.0029661 PMC3256170 22253754

[49] Corringer PJ, LeNoveÁre N, Changeux JP (2000) Nicotinic receptors at the amino-acid level. Annu Rev Pharmacol Toxicol 40: 431–458. 10836143 10.1146/annurev.pharmtox.40.1.431

[50] Arias HR (2000) Localization of agonist and competitive antagonist binding sites on nicotinic acetylcholine receptors. Neurochem Int 36: 595–645. 10771117 10.1016/s0197-0186(99)00154-0

[51] Dennis M, Giraudat J, Kotzyba-Hibert F, Goeldner M, Hirth C, et al (1988) Amino acids of the Torpedo marmorata acetylcholine receptor alpha subunit labeled by a photoaffinity ligand for the acetylcholine binding site. Biochemistry 27: 2346–2357. 3382627 10.1021/bi00407a016

[52] Galzi JL, Revah F, Black D, Goeldner M, Hirth C, et al (1990) Identification of a novel amino acid alpha-tyrosine 93 within the cholinergic ligand binding sites of the acetylcholine receptor by photoaffinity labeling. Additional evidence for a three loop model of the cholinergic ligands-binding sites. J Biol Chem 265: 10430–10437. 2355008

[53] Fu DX, Sine SM (1994) Competitive antagonists bridge the alpha-gamma subunit interface of the acetylcholine receptor through quaternary ammonium-aromatic interactions. J Biol Chem 269: 26152–26157. 7929328

[54] D'Andrea MR, Nagele RG (2006) Targeting the alpha7 nicotinic acetylcholine receptor to reduce amyloid accumulation in Alzheimer's disease pyramidal neurons. Curr Pharm Des 12: 677–684. 16472157 10.2174/138161206775474224

[55] Martin-Ruiz CM, Court JA, Molnar E, Lee M, Gotti C, et al (1999) Alpha4 but not alpha3 and alpha7 nicotinic acetylcholine receptor subunits are lost from the temporal cortex in Alzheimer's disease. J Neurochem 73: 1635–1640. 10501210 10.1046/j.1471-4159.1999.0731635.x

[56] Gotti C, Moretti M, Bohr I, Ziabreva I, Vailati S, et al (2006) Selective nicotinic acetylcholine receptor subunit deficits identified in Alzheimer's disease, Parkinson's disease and dementia with Lewy bodies by immunoprecipitation. Neurobiol Dis 23: 481–489. 16759874 10.1016/j.nbd.2006.04.005

[57] Dziewczapolski G, Glogowski CM, Masliah E, Heinemann SF (2009) Deletion of the alpha 7 nicotinic acetylcholine receptor gene improves cognitive deficits and synaptic pathology in a mouse model of Alzheimer's disease. J Neurosci 29: 8805–8815. 19587288 10.1523/JNEUROSCI.6159-08.2009 PMC2753494

[58] Tayeb HO, Murray ED, Price BH, Tarazi FI (2013) Bapineuzumab and solanezumab for Alzheimer's disease: is the ‘amyloid cascade hypothesis’ still alive? Expert Opin Biol Ther 2013 Apr 10. [Epub ahead of print] 10.1517/14712598.2013.789856 23574434

[59] Ho PL, Raw I (1992) Cyclic AMP potentiates bFGF-induced neurite outgrowth in PC12 cells. J Cell Physiol 150: 647–656. 1311333 10.1002/jcp.1041500326

[60] Xiao Y, Meyer EL, Thompson JM, Surin A, Wroblewski J, et al (1998) Rat alpha3/beta4 subtype of neuronal nicotinic acetylcholine receptor stably expressed in a transfected cell line: pharmacology of ligand binding and function. Mol Pharmacol 54: 322–333. 9687574 10.1124/mol.54.2.322

[61] Krivoshein AV, Hess GP (2004) Mechanism-based approach to the successful prevention of cocaine inhibition of the neuronal (alpha 3 beta 4) nicotinic acetylcholine receptor. Biochemistry 43: 481–489. 14717603 10.1021/bi034838l

[62] Niu L, Abood LG, Hess GP (1995) Cocaine: mechanism of inhibition of a muscle acetylcholine receptor studied by a laser-pulse photolysis technique. Proc Natl Acad Sci U S A 92: 12008–12012. 8618833 10.1073/pnas.92.26.12008 PMC40285

[63] Hess GP, Ulrich H, Breitinger HG, Niu L, Gameiro AM, et al (2000) Mechanism-based discovery of ligands that counteract inhibition of the nicotinic acetylcholine receptor by cocaine and MK-801. Proc Natl Acad Sci U S A 97: 13895–13900. 11095713 10.1073/pnas.240459497 PMC17672

