# Supplementary figures and images for: Rescue of Amyloid-Beta-Induced Inhibition of Nicotinic Acetylcholine Receptors by a Peptide Homologous to the Nicotine Binding Domain of the Alpha 7 Subtype
Source: PLoS One. 2013 Jul 22;8(7):e67194. doi: 10.1371/journal.pone.0067194 (PMC3718777; doi:10.1371/journal.pone.0067194)

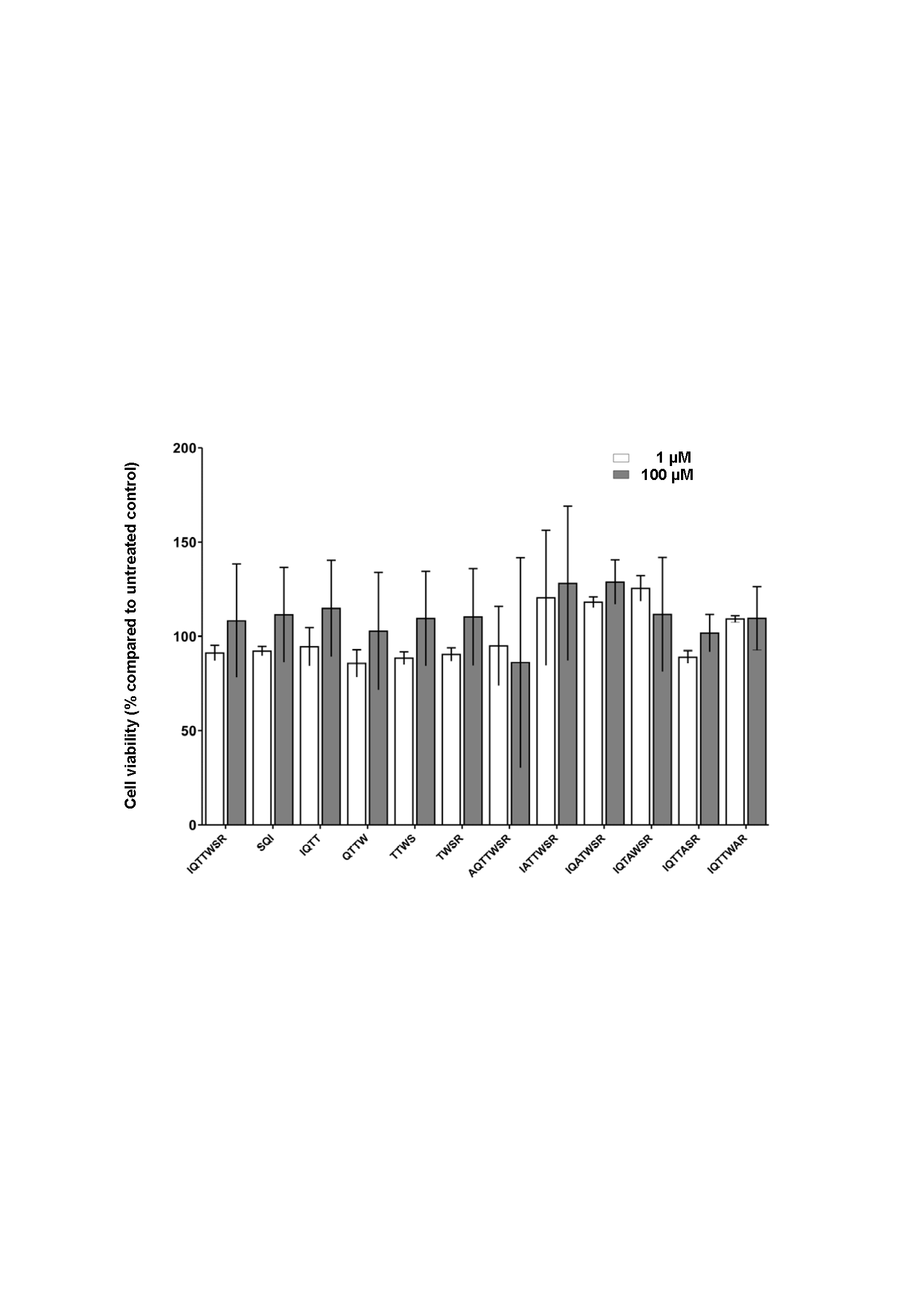

Supplement: Figure S1 — Cytotoxicity assay selected IQ analogues. PC12 cells induced to neuronal differentiation were incubated in the presence of different peptides for 48 hours, washed with PBS and stained with trypan blue. The percentages of live and dead cells of five fields per well were counted and compared to those of control cells incubated in the absence of peptides. (TIFF) [file pone.0067194.s001.tiff]

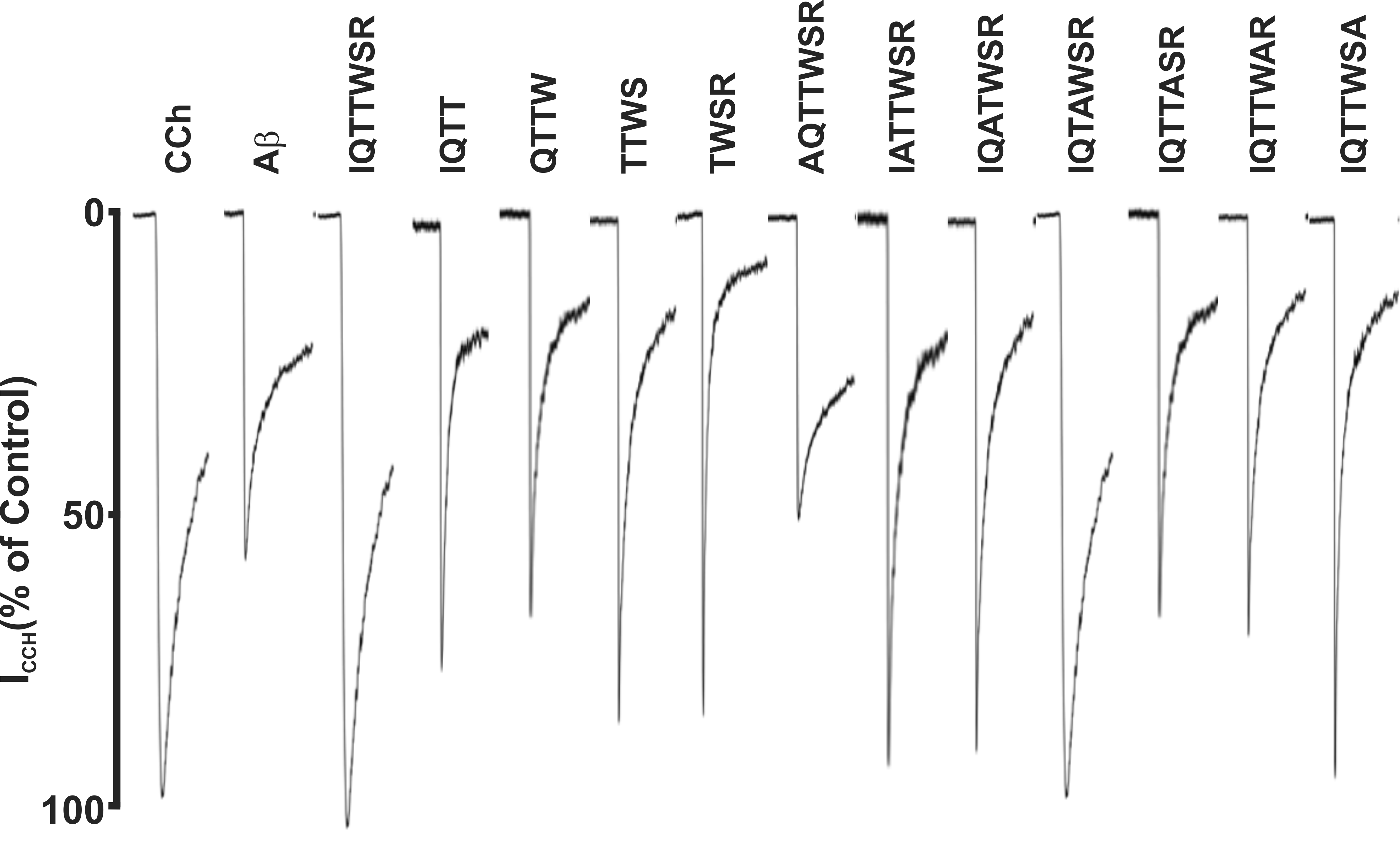

Supplement: Figure S2 — Current traces of different peptides tested for reversion of α3β4 nAChR inhibition by Aβ40. Current responses (normalized by the maximal current evoked by 0.2 mM CCh) of neuronal differentiated PC12 cells exposed for 2 s to 0.2 mM CCh plus 200 nM Aβ40 in all experimental conditions, except for the control measurement with CCh alone, and, as indicated, 500 nM of different IQ analogues. The here shown original data are illustrative for mean values ± S.D. reported in Fig. 1. (TIF) [file pone.0067194.s002.tif]

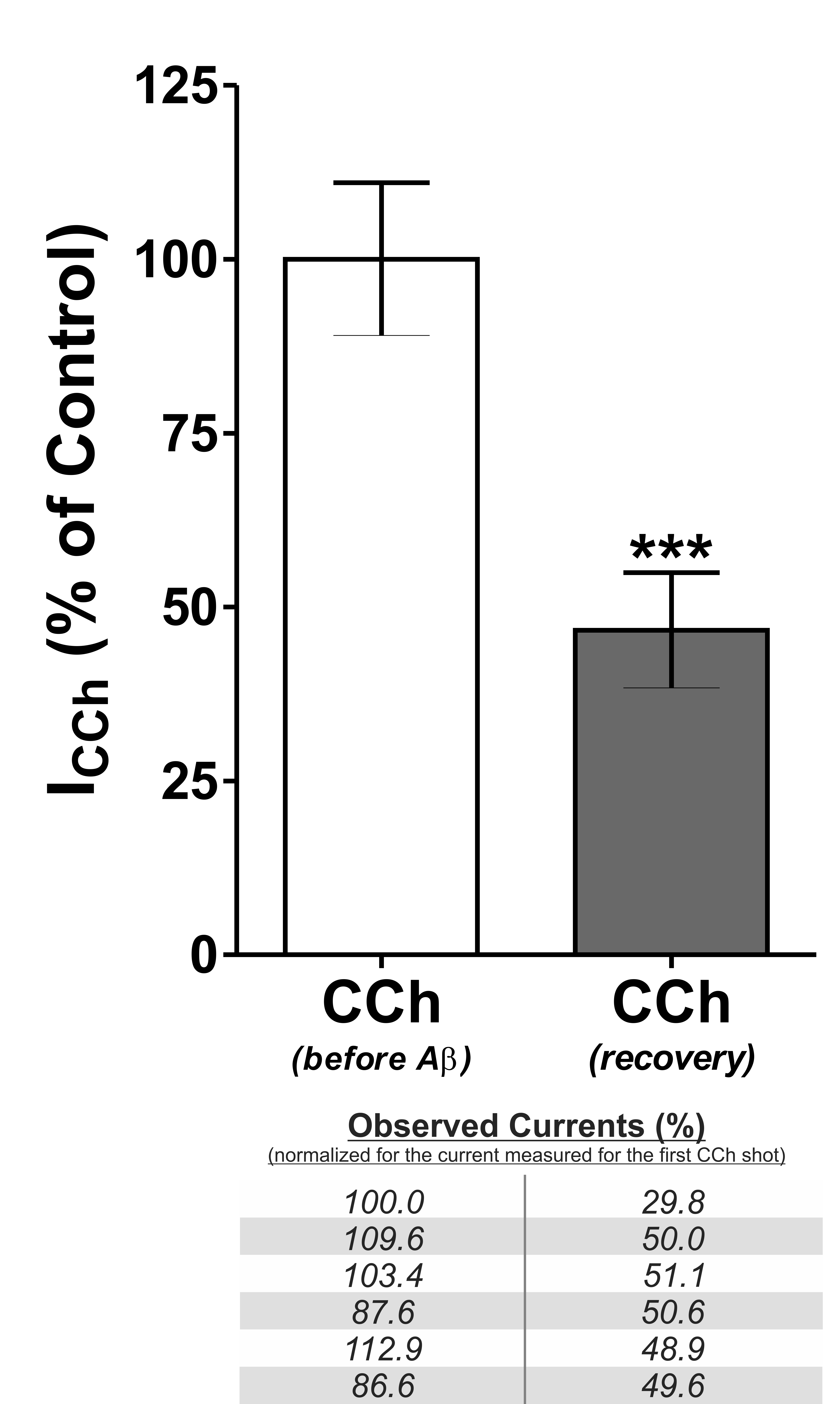

Supplement: Figure S3 — Aβ40-induced inhibition of α3β4 nAChR currents in transformed HEK cells persists after washout. Following six consecutive applications of 0.2 mM CCh, 0.2 mM CCh was co-applied once in the presence of 200 nM Aβ40. Following washout of Aβ, inhibition persisted in six consecutive applications of CCh (p<0.005, when compared to control currents measured prior to Aβ administration). (TIF) [file pone.0067194.s003.tif]

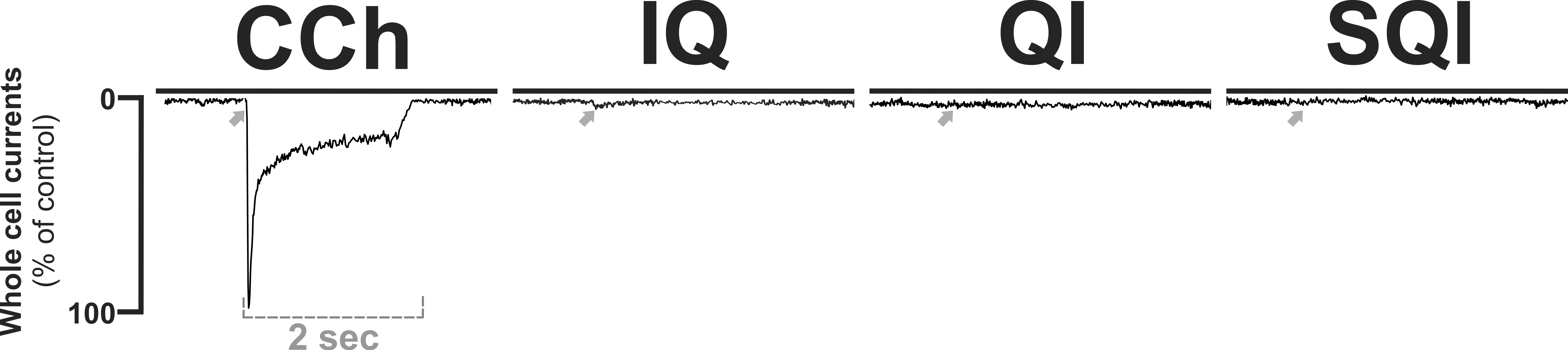

Supplement: Figure S4 — IQ, QI or SQI alone do not instigate nAChR currents in PC12 cells. The initial whole-cell response induced by 0.2 mM CCh was normalized to 100% of activity. None of the peptides (IQ, QI, SQI, tested at 2 µM) induced changes in CCh-evoked currents nor activated receptor responses in the absence of agonist. Arrows indicate time points of ligand application. (TIF) [file pone.0067194.s004.tif]
